# Supplementary material for: Enhanced rabies surveillance in roadkill specimens by real-time RT-PCR
Source: PLoS Negl Trop Dis. 2025 Jul 18;19(7):e0013348. doi: 10.1371/journal.pntd.0013348 (PMC12324673; doi:10.1371/journal.pntd.0013348)
Supplement: S1 Text — (DOCX) [file pntd.0013348.s005.docx]

**FIELD PROTOCOL for ANIMAL SAMPLE COLLECTION for RT-PCR TESTING**

# Introduction:

Roadkill specimens are an important source of samples for enhanced rabies surveillance (ERS) in targeted areas where other forms of ERS sample collection may not be practical. However, when temperatures and vehicle traffic increases, the quality of roadkill specimens is unpredictable and, in many circumstances, unsatisfactory for dRIT or DFA testing due to rapid degradation of condition. The use of molecular diagnostic methods such as real-time reverse transcriptase polymerase chain reaction (RT-PCR) testing holds promise for rabies testing when other options are not practical due to poor sample quality. The USDA, APHIS, Wildlife Services, National Rabies Management Program (NRMP) is working cooperatively with the Centers for Disease Control and Prevention (CDC) to conduct a preliminary evaluation of RT-PCR methods for roadkill specimens collected from several states as part of ERS activities.

# Objective:

To determine if RT-PCR testing can detect rabid animals from very poor, extremely degraded brainstem samples (in this study, roadkills).

# Safety:

All people involved in sample collection for rabies diagnostics must have rabies pre-exposure immunization (required by NRMP) and follow the biosafety guidelines at [https://www.cdc.gov/biosafety/index.htm.](https://www.cdc.gov/biosafety/index.htm) Personal protection is required to safely remove the brain and manipulate the tissue during sample collection.

# Personal Protective Equipment:

Handling specimens requires disposable rubber/nitrile gloves. Cut resistant gloves and eye protection (goggles/facemask/sunglasses) are also recommended.

# Materials/Field Equipment:

- Use all the same materials you would normally use for collecting ERS brainstem samples (scalpels, specimen containers, etc.)
- In addition, you may need disposable plastic pipettes.
- You will also need a camera (smart phone, flip phone, GPS unit with camera, digital camera).

# Samples of Interest, Sample Size and Location of Samples

Between July 1, 2018, and December 31, 2021, the NRMP will target collection of 300 roadkill specimens (in total) from the following states: AL, AZ, GA, ME, NC, NY, OH, TN, VA and WV. These samples should be in very poor condition and not testable by dRIT. We are specifically looking for samples that you would normally drive by because they are so smashed, flattened, soupy or otherwise untestable by dRIT.

testing. Please do not submit intact road kills that have already been tested by dRIT as part of this study. Ideally, samples should be collected from areas of known enzootic or epizootic wildlife rabies transmission (providing a better opportunity to detect a rabid animal via RT-PCR testing). The following procedures are intended to collect samples for DFA and molecular rabies diagnostic testing (RT-PCR) by CDC.

- When possible, collect brainstem tissue following normal NRMP sample collection protocols for dRIT or DFA testing, and collect adequate tissue to test by both DFA and RT-PCR (about the size of a pea).
- If the brainstem or gross anatomy cannot be identified due to poor specimen quality, collect available brain tissues around the brainstem region using a disposable scalpel.
- If brain material has degraded to the point of liquefaction, attempt to collect sample matter using a disposable, plastic pipette.
- If the specimen is flattened and dried, but the anatomical features of the cranial cavity can still be identified, collect any material consistent with brain and cranial cavity.
- Label all samples with animal ID number (barcodes if you use them for ERS) and store samples at or below -20°C until shipment to CDC.

# Data Collection and Submission

- Take a photograph of the roadkill prior to manipulation. Include either the name of the file on the CDC datasheet or type the NRMP animal ID number on the photo.
- Complete a CDC datasheet for each sample collected (see below).
- Record collection date, state, county, species and NRMP animal ID number in a metadata sheet. Optional fields include animal sex and relative age (adult or juvenile).
- Ship frozen brain tissue samples overnight to the CDC

**Sample RNA Extraction and Testing at CDC:**

- Submitted samples will be extracted using column based manual kits or robot RNA extraction protocol.
- Extracted RNA samples will be tested by two tests for sample quality (beta-actin real-time RT- PCR assay) and presence of rabies viral RNA (the pan-lyssavirus real-time RT-PCR assay LN34).

**CDC DATASHEET for RT-PCR ROADKILL STUDY with USDA**

***Please check only one box for each question.***

# Animal ID Number:

**Current Weather Conditions:** □ Sunny □ Overcast □ Rain □ Other:

**Current Temperature:** Fahrenheit

**Previous Day’s High Temperature:** Fahrenheit

| **Type of Road Sample was Found On:** □ Interstate | □ State highway | □ County highway |
| --- | --- | --- |
| □ City street | □ Dirt road | □ Other: |

**Photo File Name:** (*only include file name if you didn’t write Animal ID# on photo*)

**Skull Condition:** □ Intact □ Fractured, but recognizable □ Severely fractured

□ Flattened □ No structures recognizable □ Other:

**Brain Condition:** (*use the same guidelines used for classifying dRIT samples*)

□ Good □ Fair □ Poor □ Very poor

# Estimated Number of Days in the Field:

□ <1 day □ 1-3 days □ >3 days □ unknown (*can’t make a guess*)

**If Not a Roadkill, Mode of Death:** (*do not fill out if sample is a road kill*)

□ Found dead □ Gunshot □ unknown (*can’t make a guess*) □ Other:

**Additional Comments (Optional):**
