# Supplementary material for: Enhanced rabies surveillance in roadkill specimens by real-time RT-PCR
Source: PLoS Negl Trop Dis. 2025 Jul 18;19(7):e0013348. doi: 10.1371/journal.pntd.0013348 (PMC12324673; doi:10.1371/journal.pntd.0013348)
Supplement: Attachment — Submitted filename: ResponsetoReviewers.pdf [file pntd.0013348.s008.pdf]

Response to Reviewers

We thank the reviewers for their time and effort in reviewing our revised manuscript. Please find our response to reviewer comments below in italic font.

Reviewers' comments:

Reviewer's Responses to Questions

Key Review Criteria Required for Acceptance?

As you describe the new analyses required for acceptance, please consider the following:

Methods

-Are the objectives of the study clearly articulated with a clear testable hypothesis stated?

-Is the study design appropriate to address the stated objectives?

-Is the population clearly described and appropriate for the hypothesis being tested?

-Is the sample size sufficient to ensure adequate power to address the hypothesis being tested?

-Were correct statistical analysis used to support conclusions?

-Are there concerns about ethical or regulatory requirements being met?

Reviewer #1: The objective of the study design is well articulated and appropriately addressed it. The sample population is clearly described and the hypotheses being tested is appropriate as well. The sample size is also sufficient to ensure and address the hypotheses being tested.The correct statistical analysis was used.

Reviewer #2: See below Summary and General Comments.

Results

-Does the analysis presented match the analysis plan?

-Are the results clearly and completely presented?

-Are the figures (Tables, Images) of sufficient quality for clarity?

Reviewer #1: Results are clearly and completely presented matching the analysis plan.

Reviewer #2: See below Summary and General Comments.

Conclusions

-Are the conclusions supported by the data presented?

-Are the limitations of analysis clearly described?

-Do the authors discuss how these data can be helpful to advance our understanding of the topic under study?

-Is public health relevance addressed?

Reviewer #1: The conclusion of the study was supported by the data analyzed and presented. The authors have also discussed to advance the understanding of the study subject and topic.

Reviewer #2: See below Summary and General Comments.

Editorial and Data Presentation Modifications?

Use this section for editorial suggestions as well as relatively minor modifications of existing data that would enhance clarity. If the only modifications needed are minor and/or editorial, you may wish to recommend “Minor Revision” or “Accept”.

Reviewer #1: Accept as it is.

Reviewer #2: See below Summary and General Comments.

Summary and General Comments

Reviewer #1: Regardless of the adequate number of sample size the positive samples were with insignificant number. There is no duplication of publication. No concern of research ethics and publication ethics.

We thank the reviewer for their time.

Reviewer #2: This manuscript is well organized and well written and provides strong evidence for their claims. The data support that RT-PCR (LN34) is more sensitive than DFA or DRIT in detecting rabies virus RNA in suboptimal (deteriorated or decomposed) brain samples and incorporation of RT-PCR

testing of roadkill animals into ERS efforts can enhance case detection rates and offers practical guidance from cost-benefit analysis of collecting and testing roadkill samples using different degradation score thresholds. Overall, this study is an important contribution by proposing a viable strategy that complements current rabies surveillance methods by providing rabies surveillance data on important wildlife reservoirs in areas where passive public health surveillance data are missing and enhance wildlife surveillance in critical areas to support both regional and national rabies management programs.

Upon initial review, some minor modifications were recommended in the methods, results and discussion sections. The authors responded to all recommended modifications enhancing clarity and correcting minor errors. I have no further suggested edits or changes to the manuscript and recommend that it be accepted for publication.

We thank the reviewer for their time.

Journal Requirements:

1) Please remove your responses to the Journal Requirements from your Cover Letter.

This has been removed.

2) We do not publish any copyright or trademark symbols that usually accompany proprietary names, eg ©, ®, or TM (e.g. next to drug or reagent names). Therefore please remove all instances of trademark/copyright symbols throughout the text, including:
- TM on page: 5.

This has been removed.

3) When completing the data availability statement of the submission form, you indicated that you will make your data available on acceptance. We strongly recommend all authors decide on a data sharing plan before acceptance, as the process can be lengthy and hold up publication timelines. Please note that, though access restrictions are acceptable now, your entire data will need to be made freely accessible if your manuscript is accepted for publication. This policy applies to all data except where public deposition would breach compliance with the protocol approved by your research ethics board. If you are unable to adhere to our open data policy, please kindly revise your statement to explain your reasoning and we will seek the editor's input on an exemption. Please be assured that, once you have provided your new statement, the assessment of your exemption will not hold up the peer review process.

Pending data have been submitted to NCBI databases.
