# Supplementary material for: Enhanced rabies surveillance in roadkill specimens by real-time RT-PCR
Source: PLoS Negl Trop Dis. 2025 Jul 18;19(7):e0013348. doi: 10.1371/journal.pntd.0013348 (PMC12324673; doi:10.1371/journal.pntd.0013348)
Supplement: Attachment — Submitted filename: ResponsetoReviewers.docx [file pntd.0013348.s007.docx]

Apologies for the delays in finishing the reviews of your manuscript. Both reviewers felt that your manuscript was a valuable contribution to the literature on rabies management. Both also had comments and suggestions for improving the manuscript. Please consider these comments, edit your manuscript accordingly and provide a point-by-point response to each of the reviewer comments. We look forward to seeing your revised manuscript and comments to reviewers.

We thank the reviewers for their time and effort spent reviewing the original submission. We have worked to respond to and address all concerns in this revised manuscript and hope to hear from you shortly regarding a final decision.

Reviewer's Responses to Questions

<b>Key Review Criteria Required for Acceptance?</b></br></br> As you describe the new analyses required for acceptance, please consider the following:</br></br>

<b>Methods</b></br></br>

-Are the objectives of the study clearly articulated with a clear testable hypothesis stated?</br> -Is the study design appropriate to address the stated objectives?</br> -Is the population clearly described and appropriate for the hypothesis being tested?</br> -Is the sample size sufficient to ensure adequate power to address the hypothesis being tested?</br> -Were correct statistical analysis used to support conclusions?</br> -Are there concerns about ethical or regulatory requirements being met?</br></br>

Reviewer #1: The objective of the study clearly aligned with stated hypothesis and the study design is appropriate to address the objective.the sample population is also appropriate and well described to test the hypothesis Sufficient sample size were also collected and analysed to support the conclusion of the study and I have no concern on the ethical and regulatory requirements.

We thank the reviewer for their time.

Reviewer #2: The aim of this study is to evaluate the effectiveness of an enhanced rabies surveillance (ERS) strategy that tests roadkill samples by real-time RT-PCR (i.e., the LN34 assay). The authors point out that most data contributing to annual rabies surveillance in the U.S. is associated with passive surveillance programs in public health, in which the focus of laboratory testing for rabies is to manage potential rabies exposures in humans, pets and peridomestic animals. These data may lead to a biased understanding of the prevalence, distribution, and epizootiology of rabies within a given region because the public health focus generally leave gaps in our understanding of rabies circulation in wildlife in regions where fewer humans reside. The authors state that targeted active surveillance (i.e., ERS strategies) can expand our understanding of the prevalence and geographic distribution of wildlife rabies, thus improving rabies prevention and control activities, particularly in areas where active intervention programs (i.e., oral rabies vaccination) have been implemented to limit the spread of rabies. The objectives of the 4-year study were clearly stated; to evaluate the sensitivity of an enhanced sample collection/test method (i.e., the use of LN34 real-time RT-PCR test for rabies diagnosis in roadkill samples) to detect rabies cases in wildlife, to categorize samples based on likelihood of rabies detection using the LN34 RT-PCR method, and with that information, develop a multivariable logistic regression model to predict the likelihood of obtaining indeterminate results based on sample quality indicators such as skull and brain condition, temperature, and time in the field as well as perform a cost-benefit analysis of collecting and testing roadkill samples using different degradation score thresholds to provide guidance for best use of this enhanced surveillance strategy. The study design, which included collecting roadkill samples across ten states in known rabies enzootic areas and comparing this ERS method with data collected using traditional enhanced surveillance methods, is appropriate for the stated objectives. While the sample sizes were modest, they were sufficient to provide adequate power to support the hypotheses being tested. There are no concerns about this study not meeting ethical or regulatory requirements.

We thank the reviewer for their time.

--------------------

<b>Results</b></br></br>

-Does the analysis presented match the analysis plan?</br> -Are the results clearly and completely presented?</br> -Are the figures (Tables, Images) of sufficient quality for clarity?</br></br>

Reviewer #1: The result of this study was clearly and completely presented and well illustrated in tables and figures.

We thank the reviewer for their time.

Reviewer #2: The 4-year study focused on enhanced sampling of suspect rabid animals in areas of high interest for wildlife rabies management by collecting roadkill specimens that would otherwise be inappropriate for testing by traditional, gold standard surveillance tests (DFA and DRIT). These poor-quality samples were tested by real-time RT-PCR and case detection rates by this method were compared with enhanced rabies surveillance data reported to the National Rabies Surveillance System during two years of that same time period. While the sample size was modest, the study was able to support the use of real-time RT-PCR for rabies surveillance on samples that would be unsatisfactory for testing by traditional methods. Further, sample sizes were sufficient to provide adequate power to determine that this ERS strategy significantly increased case detection rates in the eastern U.S. raccoon rabies virus variant (RRVV) oral rabies vaccination (ORV) zone. Additionally, while most PCR results on suboptimal roadkill samples were indeterminate, they were able to determine that most samples categorized as fair or good produced definitive (reportable and actionable) PCR results, and based on this observation, they were able to develop a multivariable logistic regression model to assess conditions associated with either indeterminate or definitive PCR results and predict the testability of samples and a cost estimate for testing roadkill by RT-PCR. Model-derived sample degradation scores could be used to further refine sampling strategies to maximize probabilities that most samples would produce definitive results.

Data collected and analyses presented match the analysis plan and results, including tables and figures, were presented clearly.

We thank the reviewer for their time.

--------------------

<b>Conclusions</b></br></br>

-Are the conclusions supported by the data presented?</br> -Are the limitations of analysis clearly described?</br> -Do the authors discuss how these data can be helpful to advance our understanding of the topic under study?</br> -Is public health relevance addressed?</br></br>

Reviewer #1: The conclusion of this study was well supported by the data well analysed and described. The authors have discussed how helpful it is to advance the understanding of rabies surveillance in road side killed animals. The rabies surveillance work and out put is more relevant to public health and well addressed the public health concern.

We thank the reviewer for their time.

Reviewer #2: In this paper the authors explore the potential of ERS using RT-PCR methods to detect rabies virus in roadkill samples and find that this strategy can be a cost-effective way to complement public health surveillance of rabies and fill surveillance gaps, particularly in remote areas where surveillance is relatively poor. They demonstrate that RT-PCR (i.e., LN34) tests outperform gold standard DFA and DRIT tests when testing poor or deteriorated brain material. By adding RT-PCR testing of roadkill from the eastern U.S. raccoon rabies virus variant (RRVV) oral rabies vaccination (ORV) zone, they were able to significantly increase rabies case detection rates east of the ORV zone over CDRs obtained by traditional DRIT surveillance efforts.

Additionally, through development of a multivariable logistic regression model to predict the likelihood of obtaining indeterminate results based on sample quality indicators, the authors identify those samples that are more likely to lead to a definitive RT-PCR result (i.e., positive or negative versus indeterminate). This model may be a useful and practical tool to triage roadkill samples, thus yielding a higher percentage of definitive results (increasing case detection rates) while reducing the cost of the ERS effort.

We thank the reviewer for their time.

--------------------

<b>Editorial and Data Presentation Modifications?</b></br><br/> Use this section for editorial suggestions as well as relatively minor modifications of existing data that would enhance clarity. If the only modifications needed are minor and/or editorial, you may wish to recommend “Minor Revision” or “Accept”.

Reviewer #1: Accepted as it is with no minor edition and/or comment.

We thank the reviewer for their time.

Reviewer #2: Overall, the authors have performed a well-designed study and provide very useful data on the potential of ERS using RT-PCR methods to detect rabies virus in roadkill samples to improve surveillance of wildlife rabies. The data are presented in a clear manner and the conclusions drawn about the tests are reasonable and not overstated. While this study warrants publication, there are a few minor modifications that would enhance clarity.

Methods

1. On Line 78 (also discussed on lines 326-328), the authors describe the states in which roadkill samples were collected for the study and mention that samples were collected through opportunistic or formal roadkill surveys, but do not define what the difference is between these survey approaches or detail if different survey strategies might affect or bias the numbers of roadkill samples collected or the potential for detecting positive cases (e.g., were the geographic regions that were targeted the most likely regions to detect wildlife rabies under both survey approaches)?

We have added additional text to the methods section describing the survey approaches in more detail.

2. Line 89-90: The LN34 protocol in this study diverges from the published diagnostic LN34 protocol by reducing from triplicate to duplicate testing of the LN34 target and singlicate for the internal beta-actin control. From the validation studies of the diagnostic LN34 protocol, do the authors have data on the impact of reducing replicates on accurate detection of rabies virus RNA or beta actin in a run? If there is reduced sensitivity or accuracy in detections when reducing replicates, it would be helpful to state what is known.

We have found no difference by reducing replicates when the assay is performed by laboratory staff with experience in PCR and experience running the LN34 assay. We have added additional details to the methods referencing low inter-replicate variability in the LN34 assay in a previous publication. Variability between replicates increases as the assay reaches the limit of detection. For clinical samples, running triplicate is still recommended as it allows for easy detection of unacceptable performance (variability due to instrument, equipment, operator, or other issues).

3. Lines 100-101 and Table 3: For evaluating case detection rates, comparisons are made between LN34 Poor Quality Samples vs LN34 Good Quality Samples, but it is unclear how that determination was made. In evaluating brain condition, values of good, fair, poor, very poor are options. Was the “Good” vs “Poor” determination based only on brain condition or on a combination of brain condition and other factors (e.g., skull condition, temp, estimated # days in field)? If based upon brain condition, is “Good” = good and fair samples and "Poor" = poor and very poor?

We have added additional text to the methods to clarify this point.

4. Line 124 and CDC Datasheet: How does one estimate “Days in the Field” when one first encounters a roadkill specimen? Are there any guides to make that determination somewhat objective? If so, it would be helpful to describe.

5. Lines 126-127 and CDC Datasheet: Assessing some of the parameters that were used to describe the condition of samples collected in the field inevitably involves subjective judgement; however, it is not clear if there was any training or documentation provided to field collectors to attempt to standardize categorization of parameters that were checked on the “CDC datasheet for RT-PCR Roadkill Study with USDA” form and that were used to generate statistics for the study. For example, determination of brain condition as good, fair, poor, and very poor in S1 Table did not necessarily match with the physical description of the samples collected. There appeared to be similar descriptions that in some cases were categorized as good and in other cases as fair or poor. It would be very helpful to discuss any attempts to try to standardize those categorizations or discuss the limitations of not being able to standardize.

We have added additional text to the methods and discussion to clarify the methods and bring attention to this limitation caused by subjective descriptions of roadkill. We agree that training and experience are important to standardize the process as much as possible. The Wildlife Services biologists and technicians who performed the roadkill surveys had experience and were the same staff who would collect “good quality” roadkill samples for rabies testing by DRIT or DFA.

Results

6. Line 219, it is mentioned that there was no significant difference when comparing CDRs for samples designated as poor or very poor to “either good or very good quality”. Were there any samples designated as very good? That term is not listed under the criteria for modeling or in the supplemental tables.

This mistake has been corrected. Thank you, it has been changed to “good or fair”.

7. Lines 196-204: In the descriptions of the three positive samples that contained no recognizable brain tissue, particularly the first sample (i.e., leathery brown tissue) was there further investigation with the field collectors to obtain more specific details about what they targeted for collection? Despite that the samples “contained no recognizable brain tissue”, the Ct values were reasonably good, so was it thought that the samples were in fact extremely poor-quality brain tissue or it is thought that other tissues might yield positive results in the LN34 assay? Are there tissues studies that suggest alternative sample types might be useful for sampling?

Additional text was added to the discussion to address this point. Based on this study, we did not identify alternative sample types that seemed useful for sampling. While we did receive skin and muscle-like tissues, they did not produce positive results.

--------------------

<b>Summary and General Comments</b></br></br> Use this section to provide overall comments, discuss strengths/weaknesses of the study, novelty, significance, general execution and scholarship. You may also include additional comments for the author, including concerns about dual publication, research ethics, or publication ethics. If requesting major revision, please articulate the new experiments that are needed.

Reviewer #1: I think the surveillance work is impactful if extrapolated to other regions like Africa where road side killings of small and large wild animals around the National park areas is regularly occur. This information would also help to further investigate other wild life pathogens of zoonotic origin.

We thank the review for their time and effort.

Reviewer #2: This manuscript is well organized and well written and provides strong evidence for their claims. The data support that RT-PCR (LN34) is more sensitive than DFA or DRIT in detecting rabies virus RNA in suboptimal (deteriorated or decomposed) brain samples and incorporation of RT-PCR testing of roadkill animals into ERS efforts can enhance case detection rates and offers practical guidance from cost-benefit analysis of collecting and testing roadkill samples using different degradation score thresholds. Overall, this study is an important contribution by proposing a viable strategy that complements current rabies surveillance methods by providing rabies surveillance data on important wildlife reservoirs in areas where passive public health surveillance data are missing and enhance wildlife surveillance in critical areas to support both regional and national rabies management programs.

We thank the review for their time and effort.

--------------------

PLOS authors have the option to publish the peer review history of their article (<a href="<https://journals.plos.org/plosntds/s/editorial-and-peer-review-process#loc-peer-review-history>" target="_blank">what does this mean?</a>). If published, this will include your full peer review and any attached files.<br><br>

If you choose “no”, your identity will remain anonymous but your review may still be made public.<br><br>

<b>Do you want your identity to be public for this peer review?</b> For information about this choice, including consent withdrawal, please see our <a href="<https://www.plos.org/privacy-policy>" target="_blank">Privacy Policy</a>.

Reviewer #1: Yes: Asefa Deressa

Reviewer #2: No

Figure Files:

While revising your submission, please upload your figure files to the Preflight Analysis and Conversion Engine (PACE) digital diagnostic tool, <https://pacev2.apexcovantage.com>. PACE helps ensure that figures meet PLOS requirements. To use PACE, you must first register as a user. Then, login and navigate to the UPLOAD tab, where you will find detailed instructions on how to use the tool. If you encounter any issues or have any questions when using PACE, please email us at [figures@plos.org](mailto:figures@plos.org).

Figures have been uploaded to PACE as requested with no issues.

Data Requirements:

Please note that, as a condition of publication, PLOS' data policy requires that you make available all data used to draw the conclusions outlined in your manuscript. Data must be deposited in an appropriate repository, included within the body of the manuscript, or uploaded as supporting information. This includes all numerical values that were used to generate graphs, histograms etc.. For an example see here: <http://www.plosbiology.org/article/info%3Adoi%2F10.1371%2Fjournal.pbio.1001908#s5>.

Rabies virus partial genome sequences will be submitted to GenBank this week. Accession numbers will be added to the manuscript prior to publication.

Reproducibility:

To enhance the reproducibility of your results, we recommend that you deposit your laboratory protocols in protocols.io, where a protocol can be assigned its own identifier (DOI) such that it can be cited independently in the future. Additionally, PLOS ONE offers an option to publish peer-reviewed clinical study protocols. Read more information on sharing protocols at <https://plos.org/protocols?utm_medium=editorial-email&utm_source=authorletters&utm_campaign=protocols>

The basic protocol for this assay is already available via a protocols.io.

References

Please review your reference list to ensure that it is complete and correct. If you have cited papers that have been retracted, please include the rationale for doing so in the manuscript text, or remove these references and replace them with relevant current references. Any changes to the reference list should be mentioned in the rebuttal letter that accompanies your revised manuscript. If you need to cite a retracted article, indicate the article's retracted status in the References list and also include a citation and full reference for the retraction notice.

Reference list has been reviewed.
